# Epidemiological topology data analysis links severe COVID-19 to RAAS and hyperlipidemia associated metabolic syndrome conditions

**DOI:** 10.1093/bioinformatics/btae235

**Published:** 2024-06-28

**Authors:** Daniel Platt, Aritra Bose, Kahn Rhrissorrakrai, Chaya Levovitz, Laxmi Parida

**Affiliations:** IBM Research, 1101 Kitchawan Rd, Yorktown Heights, NY, 10598, United States; IBM Research, 1101 Kitchawan Rd, Yorktown Heights, NY, 10598, United States; IBM Research, 1101 Kitchawan Rd, Yorktown Heights, NY, 10598, United States; IBM Research, 1101 Kitchawan Rd, Yorktown Heights, NY, 10598, United States; IBM Research, 1101 Kitchawan Rd, Yorktown Heights, NY, 10598, United States

## Abstract

**Motivation:**

The emergence of COVID-19 (C19) created incredible worldwide challenges but offers unique opportunities to understand the physiology of its risk factors and their interactions with complex disease conditions, such as metabolic syndrome. To address the challenges of discovering clinically relevant interactions, we employed a unique approach for epidemiological analysis powered by redescription-based topological data analysis (RTDA).

**Results:**

Here, RTDA was applied to Explorys data to discover associations among severe C19 and metabolic syndrome. This approach was able to further explore the probative value of drug prescriptions to capture the involvement of RAAS and hypertension with C19, as well as modification of risk factor impact by hyperlipidemia (HL) on severe C19. RTDA found higher-order relationships between RAAS pathway and severe C19 along with demographic variables of age, gender, and comorbidities such as obesity, statin prescriptions, HL, chronic kidney failure, and disproportionately affecting Black individuals. RTDA combined with CuNA (cumulant-based network analysis) yielded a higher-order interaction network derived from cumulants that furthered supported the central role that RAAS plays. TDA techniques can provide a novel outlook beyond typical logistic regressions in epidemiology. From an observational cohort of electronic medical records, it can find out how RAAS drugs interact with comorbidities, such as hypertension and HL, of patients with severe bouts of C19. Where single variable association tests with outcome can struggle, TDA’s higher-order interaction network between different variables enables the discovery of the comorbidities of a disease such as C19 work in concert.

**Availability and Implementation:**

Code for performing TDA/RTDA is available in https://github.com/IBM/Matilda and code for CuNA can be found in https://github.com/BiomedSciAI/Geno4SD/.

**Supplementary Information:**

Supplementary data are available at Bioinformatics online.

## 1 Introduction

COVID-19 (C19) and severe C19 induced by SARS-COV-2 challenged medical services around the world. It was found that metabolic syndrome represents a highly tangled contributor to severe C19 risk (Obukhov *et al.* 2020). Therefore, severe C19 presents a unique opportunity to probe the renin–aldosterone–angiotensin system (RAAS) components of metabolic syndrome. The ACE2 receptor (Lan *et al.* 2020, Medina-Enríquez *et al.* 2020) is the point of entry for SARS-COV-2 and thus was an early focus regarding RAAS hypertension therapy risks and C19 severity (Jarcho *et al.* 2020). While RAAS is an important target of HT drugs (Ferrari 2013, Bezabih *et al.* 2021, Gressens *et al.* 2021), the question of how the RAAS system impacts C19 remains unresolved.

The effect of severe C19 hyperlipidemia (HL) has also been enigmatic with positive (Kristan *et al.* 2021), neutral (Wu *et al.* 2021), and negative (Ryan *et al.* 2021) associations reported. One study (Ryan *et al.* 2021) reported a negative severity association, and claimed to be the first study to show a protective effect of statin use on C19 severity. Another study (Wu *et al.* 2021) reported neutral risk for mortality, though they excluded controlled hyperlipidemic subjects and factors leading either to admission or mortality among diabetics (Kristan *et al.* 2021), although severity as defined here was not analyzed. HL was found protective against mortality, with statins (STAT) and fibrates suggested as possible reducers of lipids-induced inflammation. An increased risk of severe C19 with HL in large cohorts and meta studies was also observed (Atmosudigdo *et al.* 2021, Dey *et al.* 2021).

In this study, we used a unique approach for redescription-based topological data analysis (RTDA) (Karisani *et al.* 2021) of clinical records to address critical epidemiological questions. We compared RTDA with standard epidemiological analysis techniques (Platt *et al.* 2016) on data from Explorys to explore the physiology of risk factors elucidated by C19 associations, through interactions with RAAS-targeted HT drugs (Ferrari 2013) and HL interaction with other risk factors for severe C19. Redescription analysis infers logical relationships among data features using cumulants, while TDA examines the topology of these logical relationships yielding insights into possible multigenic pathways. While RTDA reveals possible multigenic pathways, we extended the strengths of RTDA by applying a method which performs cumulant-based network analysis (Bose *et al.* 2021) (CuNA) to tie these features into communities and obtain relative importance of feature associations with measures of statistical significance. To this end, we found meaningful interactions between RAAS and severe C19.

## 2 Materials and methods

### 2.1 Study design

Explorys provides analytic tools and data, including de-identified electronic health records from emergency and clinical health services, and insurance records, compliant with the Health Insurance Portability and Accountability Act (HIPAA) and the Health Information Technology for Economic and Clinical Health Act (HITECH). The data are accessed from a guest portal with strictly controlled access, with a download prohibition of any primary de-identified records from the system. The portal hosts SQL access; our data snapshot was taken 24 March 2021, then containing 55 972 084 records.

Explorys adapted the Centers for Disease Control and Prevention National Center for Health Statistics (CDC NCHS) coded racial and ethnic categories, which identifies Hispanic ethnicity primarily by country, while the proportion that reported themselves as “Hispanic” is a small fraction of the general population and were generally too small to test differential drug prescription choices or diagnoses, while African Americans (AfAm) were simply described.

**Figure 1.**
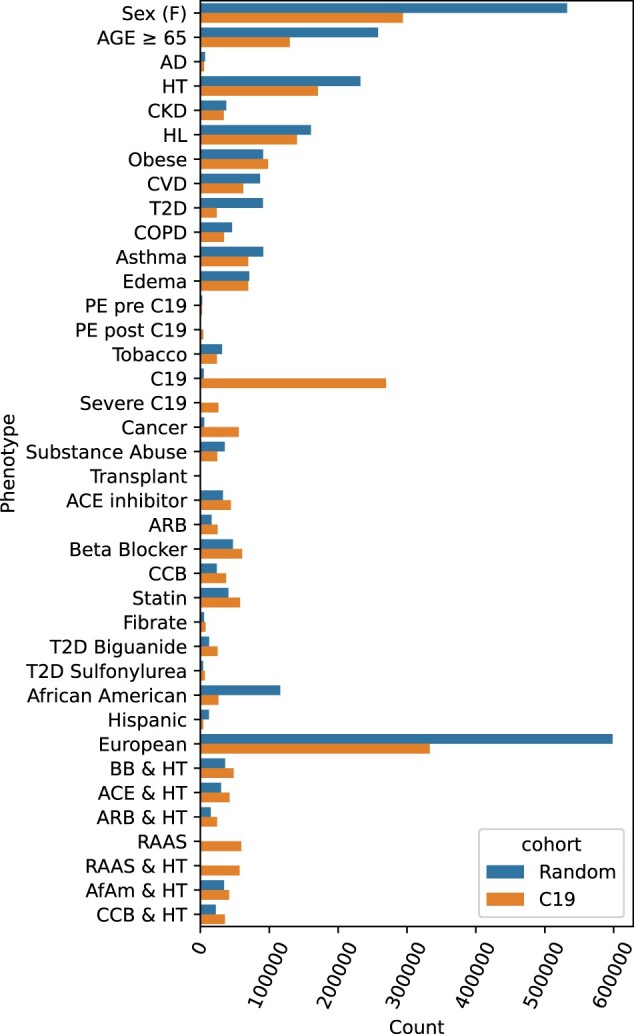
Two sample cohorts were drawn from the Explorys system: a random sample and all C19 patients plus an equal sized randomly selected non-C19 control group. The total counts indicate the number after dropping missing entries. Abbreviations include Alzheimer’s Disease (AD), hypertension (HT), chronic kidney disease (CKD), hyperlipidemia (HL), cardiovascular disease (CVD), type II diabetes (T2D), pulmonary edema (PE), beta blocker (BB), calcium channel blocker (CCB), African Americans (AfAm).

Two sets of extracted records were accessed on the following criteria. Starting with a randomly selected 1 000 000 samples, 997 140 were retained having passed missingness. Lastly, we selected all the C19 patients plus an equal number of subjects not diagnosed with C19, yielding 539 523 subjects, of which 269 536 were C19 patients (see Supplementary Table S1 for details). These splits improve statistical power where the logistic regressions (LRs) predict cases. The size of the randomly drawn set was selected to be similar in size to the C19 selected sets. The counts for relevant categories for all three sample sets are listed in Fig. 1.

### 2.2 Logistic regression

We performed logistic regression (LR) analysis using *Statsmodels* package version 0.10.2 (Seabold and Perktold 2010). We used the covariates of age (discretized as an indicator variable of individuals older than 65 considered as 1 and 0 otherwise) and sex (female as 1 and male as 0).

### 2.3 Redescription-based topological data analysis

RTDA was developed to identify logical relationships among clinical variates and evidence of multiple pathways leading to complex disease. In RTDA, inferential information is first collected by identifying significant candidate compound predicates using joint cumulants (McCullagh 2018). If the variables in the cumulant moments partition into blocks independent of each other, the joint cumulant vanishes (Percus 1975), so contributions to joint cumulants that derive entirely from products of independent lower-order cumulants cancel. Joint cumulants therefore uniquely factor out contributions from lower-order moments leaving purely *n*-way interactions. Significance was tested using Knuth’s “Algorithm P (shuffling) (Knuth 2014)” permutation tests (Fisher 1936). A null hypothesis was represented by randomly shuffled phenotype columns from which a distribution of cumulants representing randomized associations was computed against which the actual cumulants are compared. A non-significant result indicates no significant difference from higher-order correlations induced by lower-order correlations according to Percus’ theorem. This yields *P*-values and error bars associated with the null hypothesis to select significant combinations of descriptors.

Underlying biological processes and pathways constrain phenotypic relationships from which logical inferences about these processes may be constructed. For example, type II diabetes (T2D) patients have a high risk for HT. So, T2D patients are very likely to have HT, and are captured by the intersection of T2D and HT patients; therefore, T2D implies hypertension likelihood. Thus, logical implications may be derived in terms of equalities among intersections (logical “and”s) connecting these sets. These equalities are called “Redescriptions,” defined in this case as “T2D&HT.” Set inequality due to misclassification, misidentifying stages of disease progression, and other errors may be accounted for by using Jaccard distances. In the case of subsets, the Jaccard distance yields the chances that a member in the putative subset is not contained in the other set. So, clustering the joint cumulant subject sets by Jaccard distances yields logical relationships between the phenotypes implicating underlying biological processes. These approximately equal sets are called “Fuzzy Redescriptions (Parida and Ramakrishnan 2005).” This analysis relies heavily on this formulation of redescriptions. Quantifying similarity in terms of misclassification rates using Jaccard distances gives a direct connection to the error rates of derived inferences.

Several collections of joint phenotype redescription clusters connecting phenotypes to disease along multiple distinct pathways are evidence that multiple processes may lead to a pathology. We seek to recognize this situation by applying computational homology (Carlsson 2009, Edelsbrunner and Harer 2022) to identify topological loops among the pathways. The collections of simplices represent connected redescriptions. Holes among the multiple directly connected paths connecting redescriptions reflect more than one pathway to disease. Thus, topological homologies are potentially informative of complex disease processes. We applied persistent homology analysis to explore homology groups as a function of Jaccard thresholds, to characterize which redescriptions are relevant as markers along pathways identified as homological cycles (see Supplementary data). Continuous variables were thresholded at mean clinically normal value bounds. As the Jaccard filtration threshold is increased, new edges are added to the computational homology clusters. The new edges connect previously distinct diagrams. These distinct diagrams terminate at that filtration level, and a new diagram is created, often at a higher dimension, moving from homology classes H0 to H1 or H1 to H2. A topological analysis of the distances between clusters quantifies connectivity of the inferred logical statements by their misclassification rates. The network of such connections may resolve distinct pathways or etiology of disease at the cost that the ability to resolve multiple paths may be lost by allowing higher error rate connections.

### 2.4 Cumulant-based network analysis

Using CuNA, we derived a network highlighting the importance of pairwise associations across multiple sets of interactions by the number of individual joint cumulant clusters supporting the pair. For example, while HL was not itself significant to severity of C19, its interactions with other factors enhanced their impact on severity. CuNA identifies those contributions that a simple LR model might miss.

To test the significance of the CuNA network (Fig. 6), we evaluated the interactions over a range of *P*-values $P=[.01,10^{-12}]$, incrementing it by $10^{-11}$. We retained edges that occurred in all networks across different *P* thresholds (Fig. 6). The width of the edges corresponds to the number of times a pair of nodes appeared together in the cumulant groups and reflects their pairwise affinity.

We applied CuNA (Bose *et al.* 2021) to extract higher-order relationships between the RAAS drugs, disease indications, age, sex, and C19 susceptibility and severity, respectively. CuNA embeds edges connecting clinical features from each of the cumulants ranked by counts of the higher-order groups containing them, into an easily digestible network (Platt *et al.* 2016, Bose *et al.* 2021). Thereafter, meaningful functionally relevant groups are derived from community detection on statistically significant edges, clustered from the network nodes. Important interrelated modules of features, as well as the bridge nodes or communities, are tested by whether removal disconnects the networks. Their role in connecting components further highlights their key role in the shared etiology of the complex diseases. They also mark themes that emerge in redescription cycles. These were highlighted in RAAS interaction analysis.

The importance of each node is calculated as an aggregate score defined by the mean of the ranks of different network centrality measures: betweenness centrality (number of unweighted shortest paths between all pairs of nodes in the graph that passes through each node); eigenvector centrality (relative importance of a node as compared to its neighbors); degree (number of edges connected to the node); Voterank (a ranking based on a voting scheme between the neighbors of each node); and information centrality, or current flow closeness (based on effective resistance between nodes in a network).

## 3 Results

We applied LR and novel RTDA epidemiological methods on patient cohorts to identify evidence of multiple pathways leading to pathology (complex disease) or other predictive and logical relationships among variates in clinical electronic records data extracted from Explorys. Two sample sets were extracted (Fig. 1, Supplementary Table S1): random cohort (*n* = 997 140); C19 cohort (case *n* = 269 536; control *n* = 269 987); after missingness exclusions. We note that severe C19 was a very small subset of the random cohort.

### 3.1 Identifying relationships with LR

**Figure 2.**
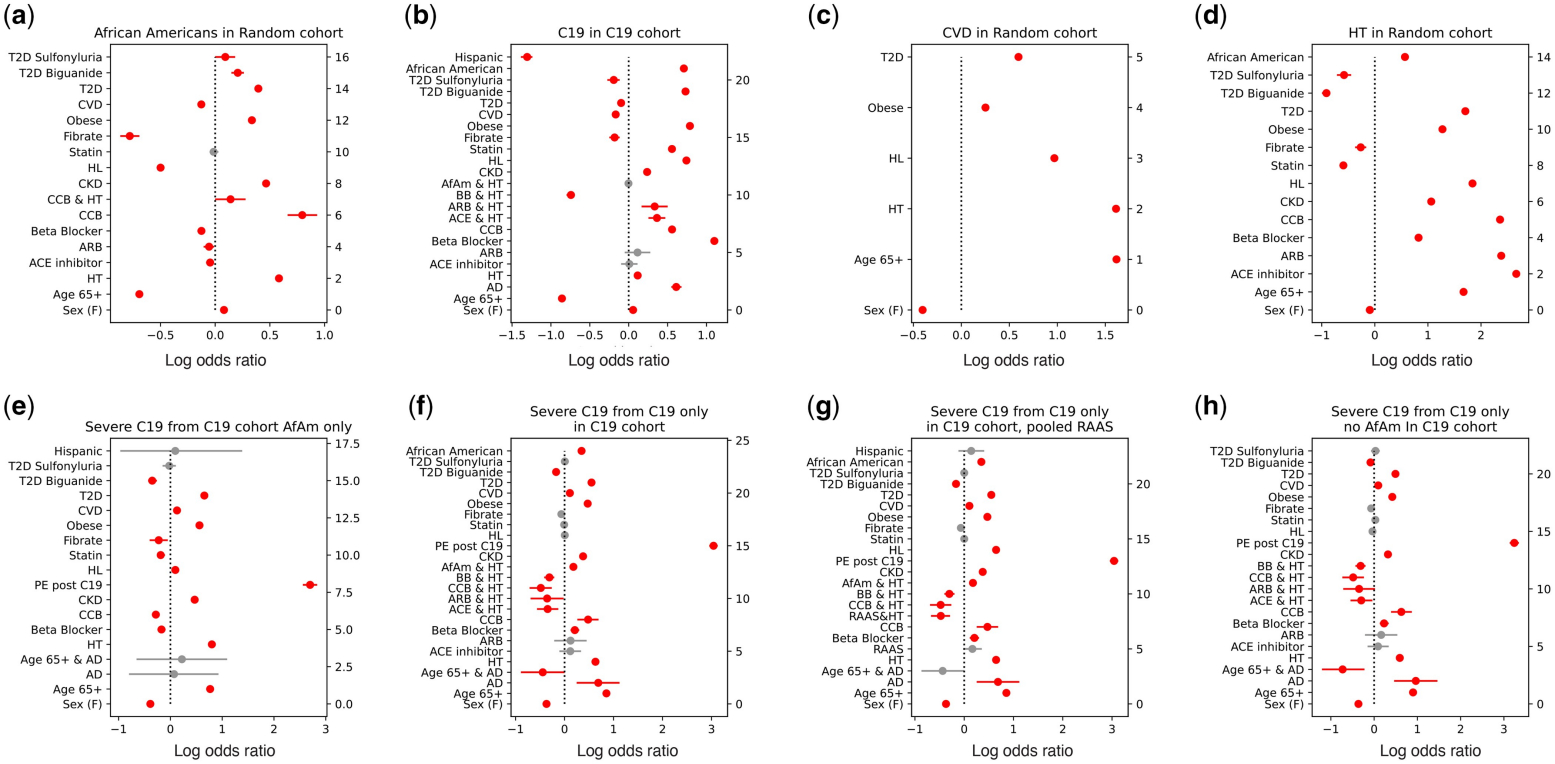
Stratified logistic regressions predicting (a) Black samples’ affinity drawn from the randomized cohort, (b) C19 infection from the C19 stratified cohort, (c) CVD in the randomized cohort, (d) HT in the randomized cohort, (e) Severe C19 in the Black C19 only cohort, (f) C19 from the C19 cohort, (g) C19 from the C19 cohort, using pooled RAAS drugs as a covariate, (h) C19 from the C19 cohort absent African Americans. Log odds ratios are plotted with 95% confidence intervals and colored red if *P*-value <.05, otherwise gray.

We first used LR to investigate the effect of RAAS on the impact of HT and severe C19; thereby using severe C19 to probe RAAS (Fig. 2 and Supplementary Fig. S1). Our LR findings (Supplementary Table S2) also establish a familiar baseline for RTDA, which can be used to validate relationships captured by RTDA and highlight novel phenotypes LR is unable to discover. We probed metabolic syndrome associated drugs including RAAS and non-RAAS drugs for HT, biguanides and sulfonylureas for T2D, and fibrates and STAT for HL. While we found HT is strongly associated with severe C19, we were able to isolate the contribution of the RAAS hypertension pathway to severe C19 by testing the interaction of HT with RAAS drugs (ACE inhibitors and ARBs), compared to non-RAAS drugs (beta blockers and calcium channel), on the association of HT with severe C19’s. Additionally, we observed distinct metabolic syndrome features among AfAm, including being prone to HT, T2D, and subsequent kidney failure (Fig. 2a). RAAS prescription use among AfAm lack power to test interactions between RAAS and hypertension on severe C19 risk, thus limiting resolution of RAAS drug influence among AfAm. The distinctive features of African American metabolic syndrome and severe C19 make this a potentially fruitful population to probe metabolic syndrome pathways.

LRs comparing the impact of RAAS and non-RAAS drugs interacting with HT in predicting severe C19 in the C19 cohort were different (Fig. 2f). The odds ratios (ORs) for ACE inhibitors and ARBs were OR = 1.497 (95% CI: 1.09–2.05, *P* = .012) and 1.492 (0.93–2.39, *P* = .0958), respectively, and for beta blockers and CCBs 1.702 (95% CI: 1.47–1.96, $P<5\times 10^{-4}$) and 1.872 (95% CI: 1.37–2.55, $P<5\times 10^{-4}$). Pooled RAAS drug recipients, RAAS drug use on hypertensive association with severe C19, was OR = 1.405 (95% CI: 1.07–1.84, *P* = .0144). The beta blockers and CCBs for that regression showed 1.738 (95% CI: 1.51–2.00, $P<5\times 10^{-4}$) and 1.897 (95% CI: 1.39–2.59, $P<5\times 10^{-4}$), respectively. The OR of hypertension by itself was 1.880 (95% CI: 1.80–1.97, $P<5\times 10^{-4}$). This suggests that treatment of hypertension with RAAS drugs may decrease risk of severe C19 compared to non-RAAS drugs.

### 3.2 Redescription-based topological data analysis

**Figure 3.**
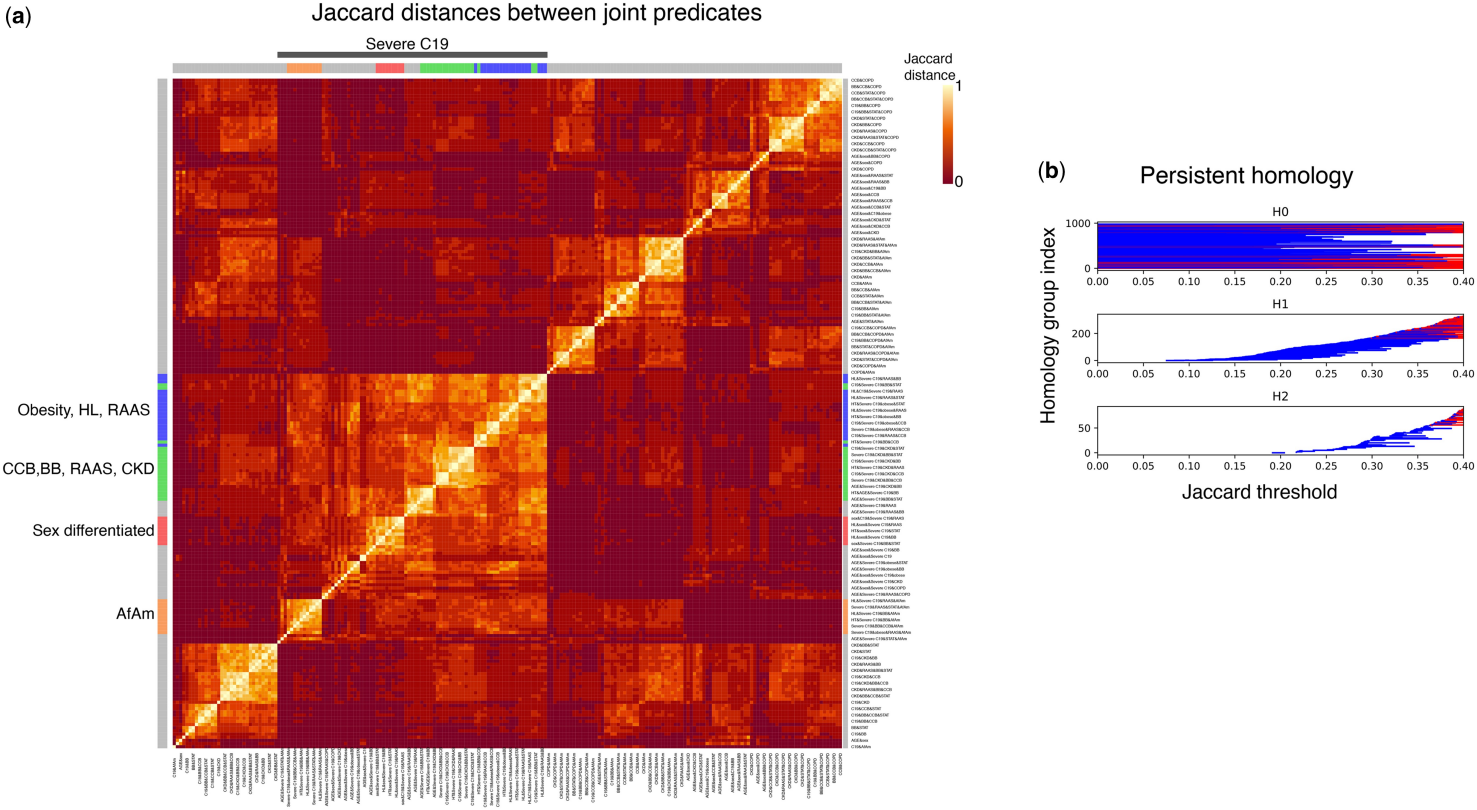
(a) Jaccard distances between joint predicates. Severe C19 cluster is indicated with a gray bar (top). The primary homologous subclusters are indicated in blue, green, red, and orange. (b) Barcode plots for the Jaccard distances shown in panel (a). Abscissa is Jaccard threshold. Ordinate is homology group index ranked by Jaccard filtration threshold. The groups H0, H1, and H2 refer to dimensions 0, 1, and 2.

RTDA analysis of these study cohorts to identify which pathways may be associated to C19 severity and RAAS found 1940 possible compound predicates. These significant compound predicates were clustered to identify 1825 (P<.05) significant joint predicates, or “redescriptions.” Fig. 3a and Supplementary Fig. S2 present a heatmap of Jaccard distances between joint predicates.

Persistent homology relationships among representative cluster connections are shown in Fig. 3b. The horizontal bars mark connected diagrams (cycles, surfaces, volumes, etc., represented as “simplices”). With an upper bound of 0.5 applied to the Jaccard distance filtration, there were 998 cycles generated in H1. The four leading cycles capturing severe C19 segregates strongly in the heatmap, and largely include most of the severe C19 cases marked by the colored sidebars (Fig. 3a).

**Figure 4.**
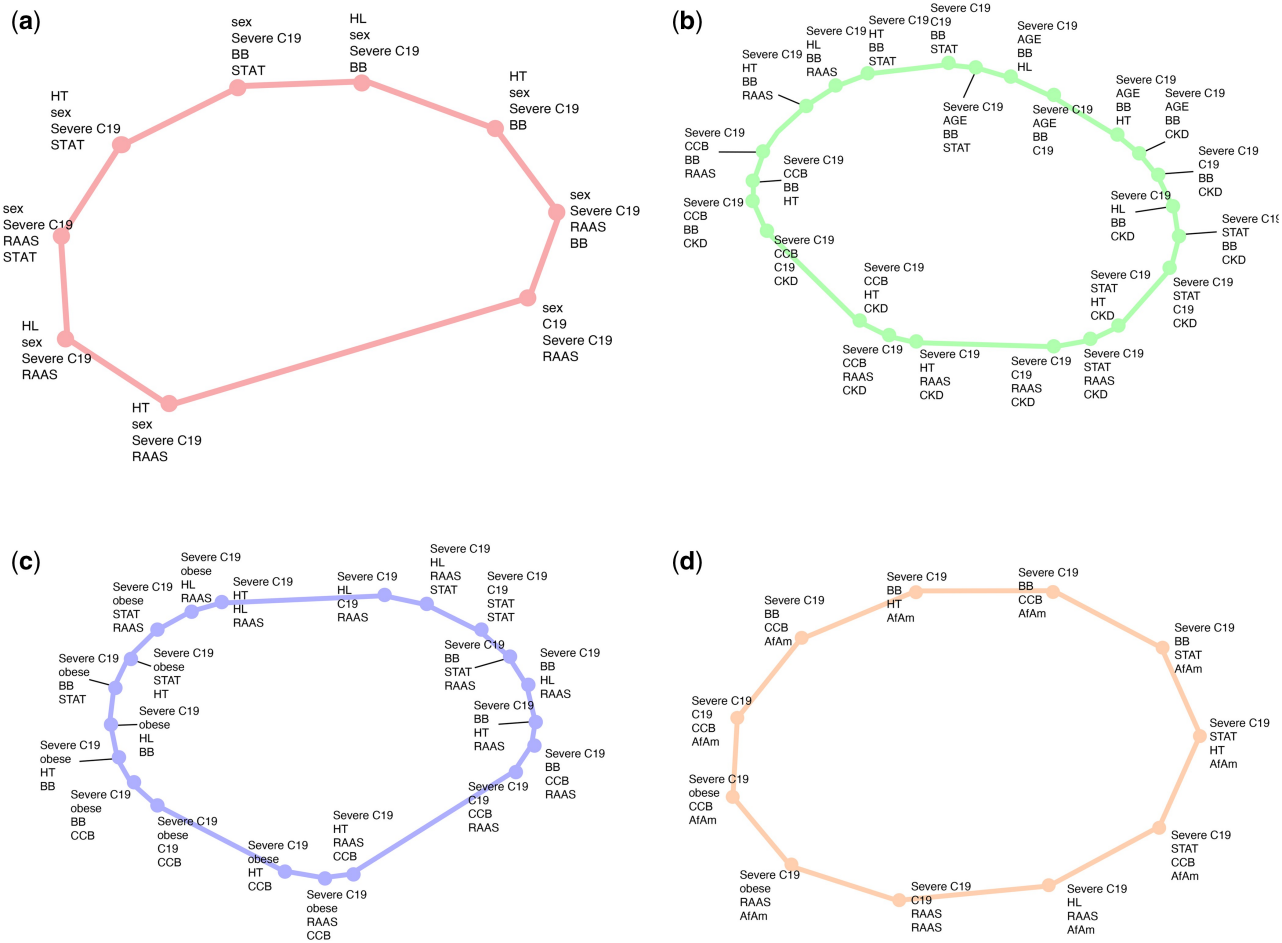
Representative cycles corresponding to side-bar color codes in Fig. 3a selected to highlight inclusions involving severe C19 and three HT drugs (ACE inhibitors, beta blockers, and ARBs), as well as African Americans. (a) ACE inhibitor cycle, (b) CCB/CKD/age cycle, (c) CCB/obesity/HL cycle, and (d) African American cycle.

Figure 4 and Supplementary Fig. S3 display some of the largest representative cycles within the homology groups identified by RTDA as highlighted among the plotted Jaccard distances (Fig. 3a). The cycles were selected to highlight inclusions involving severe C19 and three HT drugs, including ACE inhibitors, beta blockers, and ARBs, while the fourth figure involves AfAm. The largest Jaccard distance within a cycle defines the birth of the homology group bars (Fig. 3b). The largest distances in each cycle were: 0.254—ACE inhibitor cycle (Figs 3a and 4a, red); 0.370—age, chronic kidney disease (CKD), CCB, HL/statin cycle (Figs 3a and 4b, green); 0.223—CCB, obesity, and HL cycle (Figs 3a and 4c, blue); 0.321—AfAm cycle (Figs 3a and 4d, orange).

The redescription clusters tend to sit in segments connected on the larger homological cycles, suggesting multiple pathways associating these groups with each other. These clusters are:

- Red: sex differentiated responses.
- Green: CCB, BB, and RAAS together with patterns not selected by RAAS but heavily identified by other hypertensive drugs, connected to patients with chronic kidney failure with RAAS, elderly patients with RAAS, and a cluster with statin treatments.
- Blue: resembles green cycle, but with more focus on obesity and HL. It shows several sets of redescriptions connected into a multi-pathway cycle. A connecting feature of all these redescriptions is the lipids cycle—either obesity, statin prescriptions, or HL.
- Orange: AfAm with severe C19 with CCB prescription, HL, and BB prescription. Thus, RAAS components stand out as tending to be associated with obesity, HL, and statin treatment in severity of C19 in AfAm.

**Figure 5.**
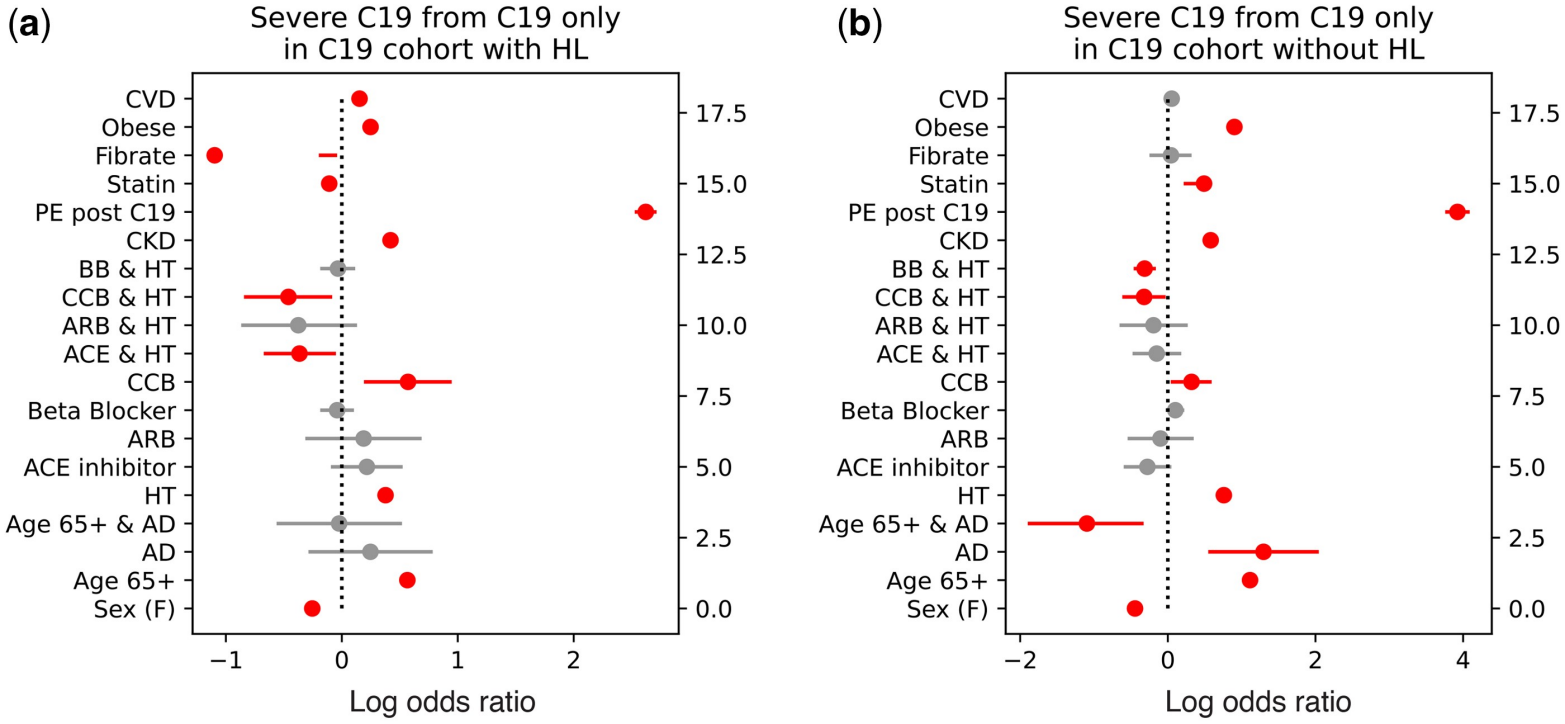
Stratified logistic regression predicting severe C19 computed on C19 only patients from the C19 cohort with (a) and without (b) hyperlipidemia. Log odds ratios are plotted with 95% confidence intervals and colored red if *P*-value <.05, otherwise gray.

Each of these cycles implicates HL as a factor among severe C19 patients, even though LR did not identify a first order association between HL and C19 severity. Our LR predicting severe C19 from the C19 patients subset in the C19 cohort while pooling ARB and ACE inhibitor RAAS drugs indicated an OR = 1.005 (95% CI: 0.97–1.04, *P* = .793) (Fig. 2g), consistent with other reports that HL is unimportant to severe C19 (Atmosudigdo *et al.* 2021, Dey *et al.* 2021, Kristan *et al.* 2021, Ryan *et al.* 2021). To understand the signals RTDA detected, we performed LRs stratified by HL (Fig. 5 and Supplementary Fig. S4). We found that HL significantly reduced the impact of other metabolic syndrome conditions on the severity of C19, including being male, age 65 or over with hypertension, chronic kidney disease, pulmonary edema during the year of the C19 infection, obesity, and type II diabetes. This protective effect is in keeping with some the published work on HL with C19. The only interaction aggravated by HL was the impact of cardiovascular disease on C19 severity (Fig. 5 and Supplementary Fig. S4).

### 3.3 Networks and communities

**Figure 6.**
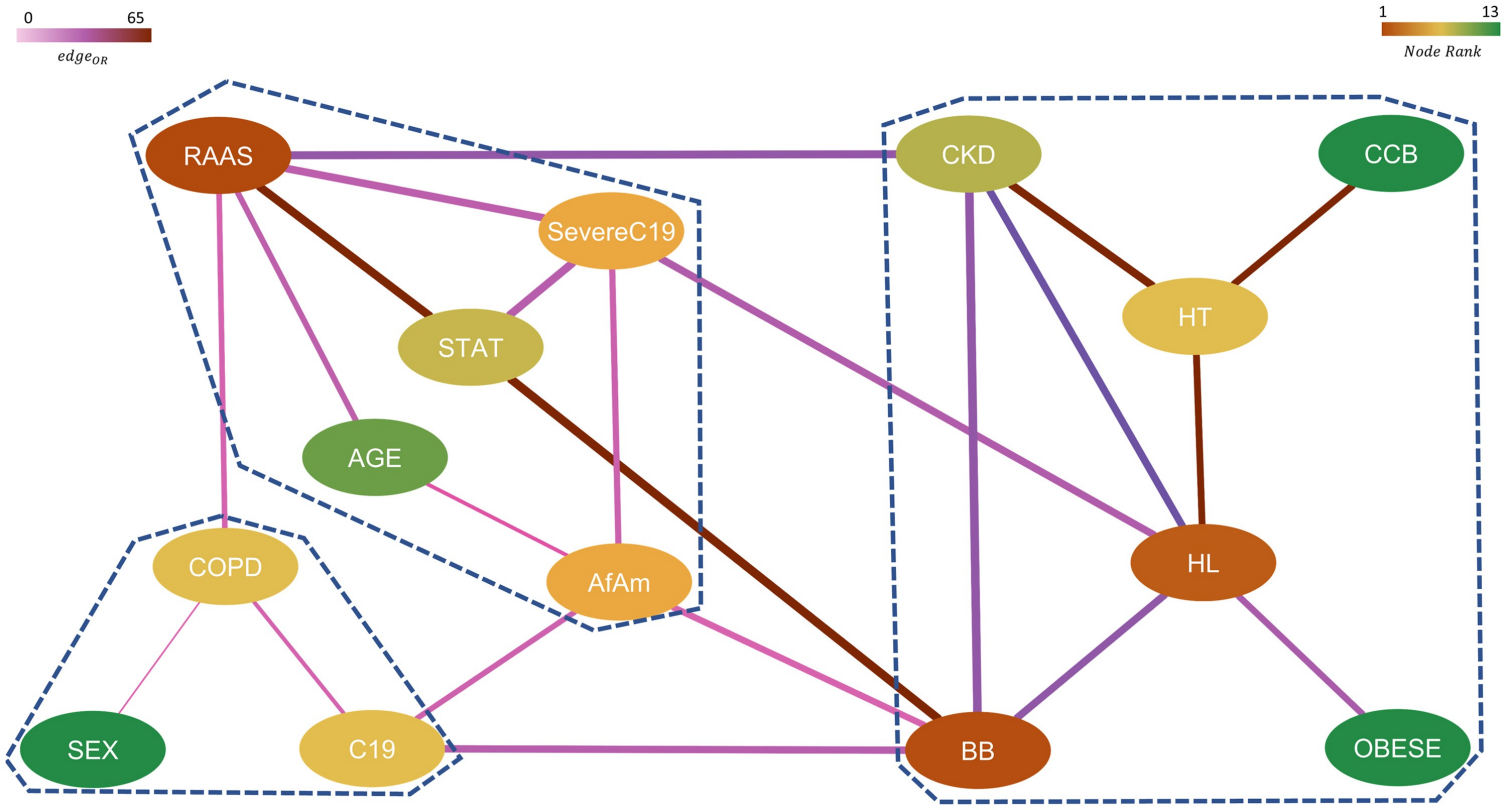
Network representing significant, higher-order interactions between different features with nodes colored by their relative rank (gradient of brown to green corresponds to higher to lower rank) and edges colored by their respective pairwise odds ratios (gradient of light to dark corresponds to low to high OR) and edge width indicates the strength of the connection between them. The communities obtained from this network are marked by dashed lines.

Using CuNA, we derived a network (Fig. 6) that highlights the importance of insightful and robust pairwise associations across various thresholds of statistical significance. We obtained 483 significant (P<.01) redescription groups from CuNA up to fourth-order interactions between 14 clinical features. These associations are often missed by a simple non-interactive LR model. CuNA discovered 22 robust edges after filtering for multiple *P*-value thresholds (as described in Section 2). CuNA highlighted RAAS as the most influential node in the network (Fig. 6) closely connected with BB, HL, AfAm, Severe C19, etc. as shown in the top five influential nodes in the network (Table 1).

**Table 1.** Top influential nodes.

Node	Aggregate	Betweenness	Eigenvector	Degree	Voterank	Information
RAAS	1.8	0.28	0.35	5	1	6.20
BB	2	0.18	0.4	5	2	6.29
HL	2.6	0.24	0.37	5	3	6.03
AfAM	5.8	0.19	0.35	4	4	5.94
Severe C19	5.8	0.09	0.34	4	6	5.87

We observed three communities from the significant cumulant groups using a greedy modularity-based algorithm (Clauset *et al.* 2004) (modularity score = 0.78). The final communities are marked by a dashed line in the CuNA network (Fig. 6).

- RAAS, severe C19, AfAm, STAT, and age.
- Beta-blockers (BB), CKD, obesity (obese), HL, HT, and CCB.
- Susceptibility to C19, chronic obstructive pulmonary disorder (COPD), and sex.

We computed and colored the edges of the network (Fig. 6) by computing the ORs between pairs of features (nodes) connected by an edge using LR (with age and sex as covariates). We observe the highest ORs are between the pairs {HT, CCB}, {HT, CKD}, {RAAS, STAT}, and {BB, STAT}. The width of an edge is denoted by the strength of the interaction between the pair of features connected by the edge. In this case, the strongest interactions were found up to fourth order within {CKD, BB}, {STAT, BB}, {STAT, RAAS}, {CKD, RAAS}, {HT, CKD}, {STAT, Severe C19}, {RAAS, Severe C19} (see Supplementary Table S3 for detailed results on network analysis).

The CuNA analysis is supported by the recovery of well-known clinical relationships. For instance, CCB and HT have the highest OR in the network as well as one of the strongest interactions up to fourth order. The community of BB, HL, and obesity follows from the fact that BB can have an adverse effect on blood lipids (Lehtonen 1985, Wolinsky 1987, Iaccarino *et al.* 2005) and obesity is a well-documented side effect of BB. RAAS and STAT drugs, along with HL are shown to have a strong interaction with severe C19 in the network. Furthermore, RAAS is also found in the same community as severe C19 and STAT. This relationship is in concordance with previous findings (Coto *et al.* 2021) where it was shown that the ACE2 gene expression, which is influenced by RAAS components, mediated by sex and age can be a primary cause of higher risk of severe C19 in older men.

## 4 Discussion

Clinical electronic medical records data can provide a view of interactions between emergent syndromes, such as C19 and complex disease comorbidities. Utilizing RTDA and CuNA, we discovered higher-order combinatorial relationships between the metabolic syndrome comorbidities and severe C19. We also probed C19 severity interactions with RAAS by considering drug impacts on severe C19 susceptibility. We observed a protective effect for severe C19 association with HT by RAAS-associated therapy, and for HL on other severe C19 risk factors, as well as detecting the impact of ethnicity on susceptibility.

Differential impact of hypertension on severe C19 between RAAS versus non-RAAS targeting medication users suggests that HT pathways involving RAAS are distinct in their contribution to severe C19. While HT drug prescription was a risk in itself for C19 patients registered in Explorys, the risk for severe C19 due to hypertension is reduced for patients under RAAS therapy compared to CCBs and beta blockers. This protective effect was also shown in prior work (Bezabih *et al.* 2021), though they report no overall direct impact of RAAS inhibitors distinct from the comorbidities. In our study we show there is a distinct interaction of RAAS drugs with the impact of hypertension on severe C19.

RTDA recovered severe C19 patients as a distinct cohort through cycles involving metabolic syndrome. Most of the severe C19 patients show involvement of multiple components of metabolic syndrome treated with multiple drug therapies. Moreover, HL is implicated in severe C19. This finding highlights the involvement of higher-order interactions since higher-order cumulants involving HL would have cancelled if the associations could have been accounted for entirely by lower-order cumulants. We verified the significant cumulants of hypertension and other metabolic syndrome components and C19 severity by interactive LR models. This is in contrast to the weak direct association between HL with C19 severity found by non-interactive analyses. The finding by RTDA of HL’s connection to severe C19 led us to revisit the role HL played that had been missed by a first order LR. An analysis of other risk factors stratified by HL showed shifts in the impact of those risk factors on C19 severity (Fig. 5 and Supplementary Fig. S4). This discovery is further corroborated by CuNA showing HL, BB, CKD, obesity, CCB, and HT appearing in the same community (Fig. 6), primarily focusing on comorbid diseases such as HT, and CKD and hypertensive drugs such as BB and CCB, all of which are strongly related to HL (Lehtonen 1985, Wolinsky 1987, Iaccarino *et al.* 2005).

While the relationship between HL and HT is expected for the general population, RTDA reveals this to be an important consideration among AfAm, where the condition tends to show lower diagnosis rates and treatments in the Explorys dataset. RTDA identifies these factors in multiple component cumulant clusters involving AfAm. While LR verified CCBs are the CPGs’ treatment of choice for AfAm and picked up other distinctive characteristics, such as lower diagnosed rate of HL, statin or fibrate prescriptions, or diagnosis of CVD, it does not adequately characterize the interconnected relationship between these factors. Furthermore, AfAm appeared among the top five most significant nodes in the CuNA network highlighting the importance to study this population in relation with C19 severity and metabolic syndromes (Fig. 6, Table 1) and it belonged to a community with elevated severity of C19. Lastly, the topological cycles and redescriptions suggested several novel relationships that could be tested by and quantified using LR to assess their validity and measure the association strengths.

There exist some natural limitations to the analysis that are intrinsic to epidemiological analyses of EHRs. In Explorys, as with other EHR repositories, health records tend to reflect reimbursement billables. Clinical tests both justify and are justified by diagnoses. For example, without a justification to order a blood panel or a test, some data are not available within a control group. This limitation dominates considerations of what tests may be available for given hypotheses using these datasets. Moreover the number of AfAm enrolled in the Explorys set, as well as the impact of CPGs on the range of hypertension pharmaceutical therapies prescribed, has limited the statistical power among AfAm for probing RAAS interactions with the associated risk of hypertension on severe C19. It is well known that racial and ethnic minorities had higher risk of severe C19 and it affected these communities disparately. Socioeconomic indicators were also found to be strongly associated with C19 for racial and ethnic minorities. The entire scope of analyzing the social and demographic determinants of disparities in severe C19 outcome can be outside of the Explorys dataset due to low sample sizes. Lastly, in a very basic way, all records refer to patients. Interchangeability between terms of “patients” and “subjects” reflects this awareness.

## 5 Conclusion

RTDA enabled the discovery of higher-order relationships beyond typical first order LR models exploring epidemiological questions. We used RTDA to take advantage of C19 to understand the relationship between the risk of the RAAS component of metabolic syndrome pathways plays with C19 severity. We found that the RAAS complex was important due to involvement of CKD, and the trend towards suppression of the association of hypertension with severe C19 by RAAS drugs versus non-RAAS drugs. Redescriptions and homological cycles did not tend to pick up such associations. Instead, metabolic syndrome and related conditions appear to be important to severe C19, but with some variations depending on comorbidities or distinctive lineages. Specifically, some variations show up within AfAm compared to other groups, which is also reflected in differences in CPGs. RAAS’s central role was further confirmed by CuNA (Fig. 6) by it being the most influential node (Supplementary Table S3). The specific community containing RAAS also includes STAT and severe C19, which is meaningful as there have been conflicting reports as to the role of HL with severe C19. This community also includes age and AfAm status, emphasizing the previously known interconnection between severe C19 with AfAm and with the elderly.

The impact of RAAS versus non-RAAS hypertension therapies in mitigating the risk that HT poses to C19 severity suggests that clinical interventions employing RAAS therapies might be preferred in the presence of wide SARS-COV-2 spread. Though we acknowledge this conclusion is subject to the limitation that the visibility of shared history that promoted selection of RAAS therapies and which might have also been relevant to reducing risk of C19 severity could confound the results. However, this study on the severity of C19 highlights the potential for topological data analysis to aid in epidemiological studies looking at specific relationships to uncover novel connections beyond the power of traditional analyses.

## Supplementary Material

btae235_Supplementary_Data

## Data Availability

Code for performing TDA/RTDA is available in https://github.com/IBM/Matilda and code for CuNA can be found in https://github.com/BiomedSciAI/Geno4SD/.
