# Supplementary material for: Epidemiological topology data analysis links severe COVID-19 to RAAS and hyperlipidemia associated metabolic syndrome conditions
Source: Bioinformatics. 2024 Jun 28;40(Suppl 1):i199–207. doi: 10.1093/bioinformatics/btae235 (PMC11211822; doi:10.1093/bioinformatics/btae235)
Supplement: btae235_Supplementary_Data [file btae235_supplementary_data.pdf]

Supplementary Table 1. Two sample cohorts were drawn from the Explorys system: a random sample and all the COVID-19 patients plus an equal sized randomly selected non-Covid-19 control group. The total counts indicate the number after dropping missing entries.

**Clinical Features**	**Random Sample**		**COVID Case - Control**	
	**Count**	**%**	**Count**	**%**
Sex (F)	532,327	53.1	293,902	54.5
AGE ≥ 65	258,082	25.9	129,708	24.0
AD	6,783	0.680	5,351	0.99
HT	232,264	23.3	170,614	31.6
CKD	37,466	3.76	33,832	6.27
HL	160,444	16.1	140,225	26.0
Obese	90,954	9.12	98,343	18.2
CVD	86,682	8.69	62,180	11.52
T2D	90,755	9.10	725,003	13.9
COPD	45,891	4.60	34,429	6.38
Asthma	91,181	9.14	69,474	12.9
Edema	70,827	7.10	69,577	12.9
Pulmonary Edema pre C19	2,806	0.281	2,478	0.459
Pulmonary Edema post C19	341	0.0342	4,078	0.766
Tobacco	31,478	3.16	24,097	4.47
COVID-19	4,824	0.486	269,536	49.96
Severe COVID-19	477	0.0478	26,168	4.85
Cancer	5,580	8.58	55,642	10.3
Substance Abuse	35,412	3.55	24,395	4.52
Transplant	145	0.0145	303	0.0561
ACE inhibitor	32,749	3.28	44,215	8.20
ARB	16,282	1.63	24,989	4.63
Beta Blocker	47,107	4.72	60,460	1.12
Calcium Channel Block	23,815	2.39	37,181	6.89
Statin	40,757	4.09	57,637	10.7
Fibrate	5,458	0.547	7,563	1.40
T2D Biguanide	12,614	1.27	25,060	4.64
T2D Sulfonylurea	3,789	0.380	6,686	1.23
African American	115,831	11.6	26,112	17.8
Hispanic	12,390	1.24	4,146	0.768
European	598,494	60.0	332,970	61.7
BB & HT	35,754	3.59	48,247	8.98
ACE & HT	30,099	3.02	42,357	7.85
ARB & HT	15,141	1.52	24,238	4.49
RAAS	--	--	59,419	11.01
RAAS & HT	--	--	56,909	11.01
AfAm & HT	34,420	3.45	41,613	7.71
CCB & HT	22,204	2.23	35,701	6.62
**Total**	**997,140**		**539,523**	

Supplementary Table 2. Results from logistic regression associating independent clinical variates with patient cohorts.

Independent variables	Predicting Severe C19 from C19 only-in C19 cohort	Predicting Severe C19 from C19 only-in C19 cohort, pooled RAAS	Predicting Severe C19 from C19 only-no AfAm in C19 cohort	Predicting Severe C19 from C19 cohort AfAm only	Predicting C19 in C19 cohort	Predicting African Americans in Random cohort	Predicting HT in Random cohort	Predicting CVD in Random cohort	Type
Sex (F)	0.692	0.691	0.696	0.679	1.058	1.084	0.91	0.668	OR
Sex (F)	0.67-0.71	0.67-0.71	0.67-0.72	0.65-0.71	1.05-1.07	1.07-1.10	0.90-0.92	0.66-0.68	CI
Sex (F)	p < 5e-4	p < 5e-4	p < 5e-4	p < 5e-4	p < 5e-4	p < 5e-4	p < 5e-4	p < 5e-4	pval
Age 65+	2.351	2.353	2.46	2.154	0.424	0.5	5.317	5.034	OR
Age 65+	2.27-2.43	2.27-2.44	2.36-2.56	2.03-2.29	0.42-0.43	0.49-0.51	5.25-5.39	4.94-5.13	CI
Age 65+	p < 5e-4	p < 5e-4	p < 5e-4	p < 5e-4	p < 5e-4	p < 5e-4	p < 5e-4	p < 5e-4	pval
AD	1.99	1.984	2.622	1.074	1.842				OR
AD	1.28-3.07	1.29-3.06	1.59-4.32	0.45-2.53	1.73-1.97				CI
AD	p < 5e-4	0.00194	p < 5e-4	p=0.8708	p < 5e-4				pval
Age 65+ & AD	0.641	0.646	0.483	1.249					OR
Age 65+ & AD	0.41-1.00	0.42-1.00	0.30-0.80	0.52-2.99					CI
Age 65+ & AD	p = 0.049	0.0525	p=0.00507	p=0.6189					pval
HT	1.88	1.91	1.81	2.227	1.122	1.792		5.01	OR
HT	1.80-1.97	1.82-2.00	1.72-1.90	2.08-2.39	1.10-1.14	1.76-1.83		4.91-5.12	CI
HT	p < 5e-4	p < 5e-4	p < 5e-4	p < 5e-4	p < 5e-4	p < 5e-4		p < 5e-4	pval
ACE inhibitor	1.124		1.095		1.008	0.955	14.35		OR
ACE inhibitor	0.90-1.40		0.86-1.40		0.907-1.12	0.92-0.99	13.67-15.07		CI
ACE inhibitor	p = 0.295		p=0.471		p = 0.876	p = 0.019	p < 5e-4		pval
ARB	1.131		1.178		1.12	0.946	10.811		OR
ARB	0.81-1.57		0.81-1.71		0.95-1.32	0.90-0.99	10.04-11.63		CI
ARB	p = 0.463		p=0.386		p = 0.176	p = 0.025	p < 5e-4		pval
RAAS		1.181							OR
RAAS		0.98-1.43							CI
RAAS		0.08224							pval
Beta Blocker	1.233	1.23	1.259	0.842	3.007	0.882	2.279		OR
Beta Blocker	1.13-1.35	1.12-1.35	1.14-1.40	0.78-0.91	2.87-3.14	0.85-0.91	2.20-2.36		CI
Beta Blocker	p < 5e-4	p < 5e-4	p < 5e-4	p < 5e-4	p < 5e-4	p < 5e-4	p < 5e-4		pval
CCB	1.616	1.602	1.876	0.755	1.746	2.217	10.573		OR
CCB	1.30-2.00	1.29-1.99	1.48-2.39	0.70-0.81	1.69-1.81	1.94-2.54	9.94-11.25		CI
CCB	p < 5e-4	p < 5e-4	p < 5e-4	p < 5e-4	p < 5e-4	p < 5e-4	p < 5e-4		pval
ACE & HT	0.708		0.743		1.438				OR
ACE & HT	0.57-0.88		0.58-0.96		1.29-1.60				CI
ACE & HT	p < 5e-4		P=0.00205		p < 5e-4				pval
ARB & HT	0.701		0.707		1.396				OR
ARB & HT	0.50-0.98		0.49-1.02		1.18-1.65				CI
ARB & HT	p =0.037		P=0.00700		p < 5e-4				pval
RAAS & HT		0.622							OR
RAAS & HT		0.51-0.75							CI
RAAS & HT		p < 5e-4							pval
CCB & HT	0.616	0.62	0.616			1.151			OR
CCB & HT	0.49-0.77	0.50-0.77	0.48-0.79			1.00-1.32			CI
CCB & HT	p < 5e-4	p < 5e-4	p < 5e-4			p = 0.047			pval
BB & HT	0.734	0.74	0.732		0.476				OR
BB & HT	0.66-0.81	0.67-0.82	0.65-0.82		0.45-0.50				CI
BB & HT	p < 5e-4	p < 5e-4	p < 5e-4		p < 5e-4				pval
AfAm & HT	1.198	1.194			0.997				OR
AfAm & HT	1.12-1.28	1.12-1.28			0.96-1.03				CI
AfAm & HT	p < 5e-4	p < 5e-4			p = 0.881				pval
CKD	1.46	1.456	1.377	1.598	1.267	1.593	2.893		OR
CKD	1.40-1.52	1.40-1.52	1.31-1.45	1.49-1.71	1.23-1.31	1.54-1.65	2.80-2.99		CI
CKD	p < 5e-4	p < 5e-4	p < 5e-4	p < 5e-4	p < 5e-4	p < 5e-4	p < 5e-4		pval

Independent variables	Predicting Severe C19 from C19 only-in C19 cohort	Predicting Severe C19 from C19 only-in C19 cohort, pooled RAAS	Predicting Severe C19 from C19 only-no AfAm in C19 cohort	Predicting Severe C19 from C19 cohort AfAm only	Predicting C19 in C19 cohort	Predicting African Americans in Random cohort	Predicting HT in Random cohort	Predicting CVD in Random cohort	Type
Pulmonary Edema post C19	20.939	20.935	25.543	14.839					OR
Pulmonary Edema post C19	19.2-22.8	19.2-22.8	23.0-28.4	12.9-17.0					CI
Pulmonary Edema post C19	p < 5e-4	p < 5e-4	p < 5e-4	p < 5e-4					pval
HL	1.005	1.91	0.963	1.1	2.1	0.607	6.292	2.636	OR
HL	0.97-1.04	1.82-2.00	0.92-1.01	1.03-1.17	2.06-2.14	0.59-0.62	6.19-6.39	2.59-2.68	CI
HL	p = 0.793	p < 5e-4	p=0.1057	p=0.00350	p < 5e-4	p < 5e-4	p < 5e-4	p < 5e-4	pval
Statin	0.992	1.001	1.029	0.829	1.744	0.986	0.553		OR
Statin	0.95-1.04	0.96-1.05	0.98-1.083	0.77-0.90	1.69-1.80	0.95-1.03	0.53-0.57		CI
Statin	p = 0.719	0.9544	p=0.279	p < 5e-4	p < 5e-4	p = 0.503	p < 5e-4		pval
Fibrate	0.937	0.935	0.934	0.798	0.834	0.458	0.767		OR
Fibrate	0.87-1.01	0.87-1.01	0.86-1.02	0.67-0.95	0.78-0.89	0.42-0.50	0.69-0.85		CI
Fibrate	p = 0.100	0.0893	p=0.1279	p = 0.00936	p < 5e-4	p < 5e-4	p < 5e-4		pval
Obese	1.604	1.6	1.522	1.753	2.197	1.399	3.574	1.286	OR
Obese	1.55-1.66	1.55-1.65	1.46-1.58	1.66-1.85	2.16-2.24	1.37-1.43	3.50-3.65	1.26-1.32	CI
Obese	p < 5e-4	p < 5e-4	p < 5e-4	p < 5e-4	p < 5e-4	p < 5e-4	p < 5e-4	p < 5e-4	pval
CVD	1.115	1.111	1.1	1.136	0.846	0.881			OR
CVD	1.07-1.16	1.07-1.15	1.05-1.15	1.06-1.22	0.83-0.87	0.86-0.90			CI
CVD	p < 5e-4	p < 5e-4	p < 5e-4	p < 5e-4	p < 5e-4	p < 5e-4			pval
T2D	1.734	1.733	1.638	1.926	0.907	1.484	5.5	1.814	OR
T2D	1.67-1.80	1.67-1.80	1.56-1.72	1.81-2.05	0.89-0.93	1.45-1.52	5.39-5.62	1.78-1.85	CI
T2D	p < 5e-4	p < 5e-4	p < 5e-4	p < 5e-4	p < 5e-4	p < 5e-4	p < 5e-4	p < 5e-4	pval
T2D Biguanide	0.842	0.847	0.92	0.704	2.076	1.229	0.402		OR
T2D Biguanide	0.80-0.88	0.80-0.89	0.86-0.98	0.65-0.77	1.98-2.17	1.16-1.30	0.37-0.43		CI
T2D Biguanide	p < 5e-4	p < 5e-4	p < 5e-4	p < 5e-4	p < 5e-4	p < 5e-4	p < 5e-4		pval
T2D Sulfonyluria	1.006	1.001	1.032	0.978	0.825	1.097	0.56		OR
T2D Sulfonyluria	0.93-1.09	0.93-1.08	0.93-1.14	0.86-1.11	0.76-0.89	1.00-1.20	0.49-0.64		CI
T2D Sulfonyluria	p = 0.888	0.9722	0.5372	p=0.7222	p < 5e-4	p = 0.046	p < 5e-4		pval
African American	1.415	1.416			2.032		1.764		OR
African American	1.34-1.50	1.34-1.50			1.99-2.07		1.73-1.80		CI
African American	p < 5e-4	p < 5e-4			p < 5e-4		p < 5e-4		pval
Hispanic		1.153		1.099	0.271				OR
Hispanic		0.89-1.50		0.38-4.00	0.25-0.29				CI
Hispanic		0.2864		p=0.8859	p < 5e-4				pval

Supplementary Table 3. Detailed results of network analysis using CuNA. The first column denotes the Nodes and their respective rank. The following columns (third to sixth) denotes the network, with their source (From) and sink (To), their strength in the fourth order interaction found by CuNA measured as the number of times they appeared together in significant redescription groups and their odds ratio computed using a logistic regression (age and sex as covariates).

**Nodes**	**Agg Rank**	**From**	**To**	**Strength**	**OR**
Severe C19	5.8	CKD	BB	79	7.584
		STAT	BB	79	21.284
		STAT	RAAS	78	29.189
COPD	7.8	CKD	RAAS	77	7.343
		HT	CKD	77	19.911
		STAT	Severe C19	77	4.33
		RAAS	Severe C19	76	3.9154
C19	7.75	CKD	HL	75	10.678
OBESE	13	C19	BB	75	4.409
SEX	13	HL	BB	75	7.641
CCB	13	HL	Severe C19	75	4.839
		HT	CCB	74	65.503
		HT	HL	73	14.497
HL	2.6	AfAm	BB	72	1.56
		OBSESE	HL	72	5.55
AfAm	5.8	AfAm	Severe C19	71	2.583
RAAS	1.8	AGE	RAAS	70	3.72
CKD	4	AfAm	C19	69	2.44
STAT	8.5	COPD	RAAS	69	1.836
HT	7.8	COPD	C19	64	1.836
BB	2	AfAm	AGE	63	0.7
AGE	10.75	COPD	SEX	44	0.97

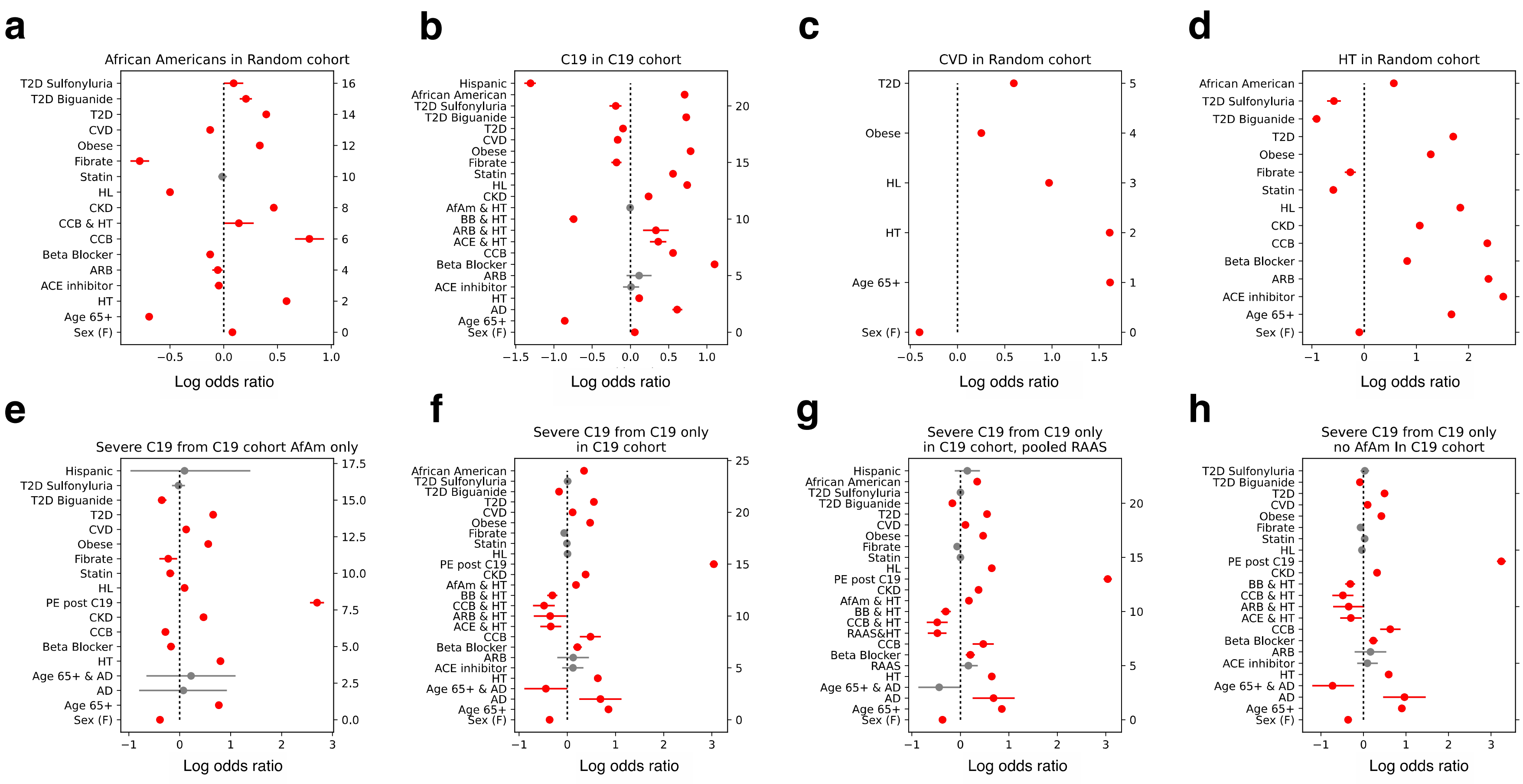

Supplementary Figure 1. Stratified logistic regressions predicting a) Black samples' affinity drawn from the randomized cohort, b) C19 infection from the C19 stratified cohort, c) CVD in the randomized cohort, d) HT in the randomized cohort, e) Severe C19 in the Black C19 only cohort, f) C19 from the C19 cohort, g) C19 from the C19 cohort, using pooled RAAS drugs as a covariate, h) C19 from the C19 cohort absent African Americans. Log odds ratios are plotted with 95\% confidence intervals and colored red if *p*-value < 0.05, otherwise gray.

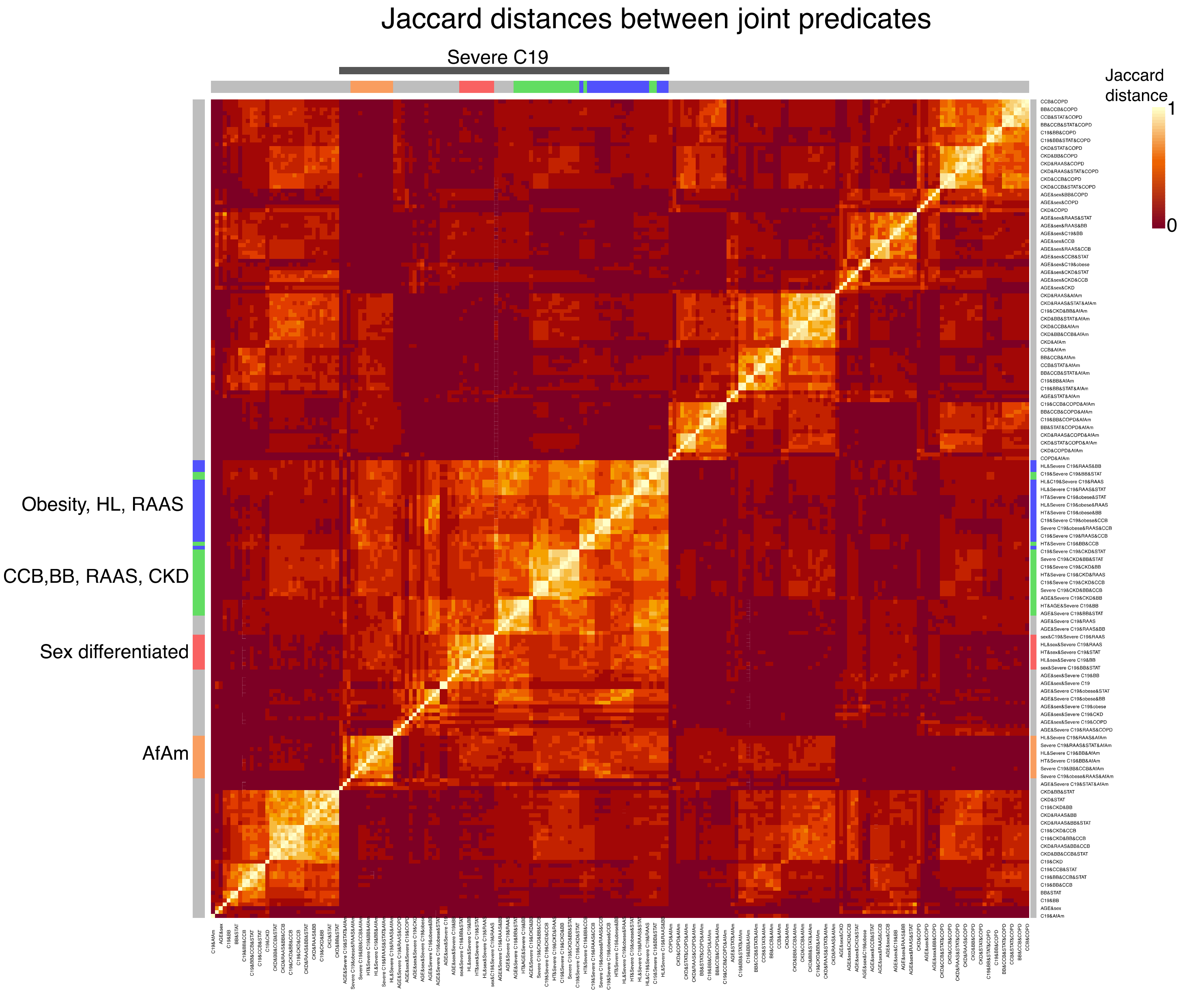

Supplementary Figure 2. Jaccard distances between joint predicates. Severe C19 cluster is indicated with a gray bar (top). The primary homologous subclusters are indicated in blue, green, red, and orange.

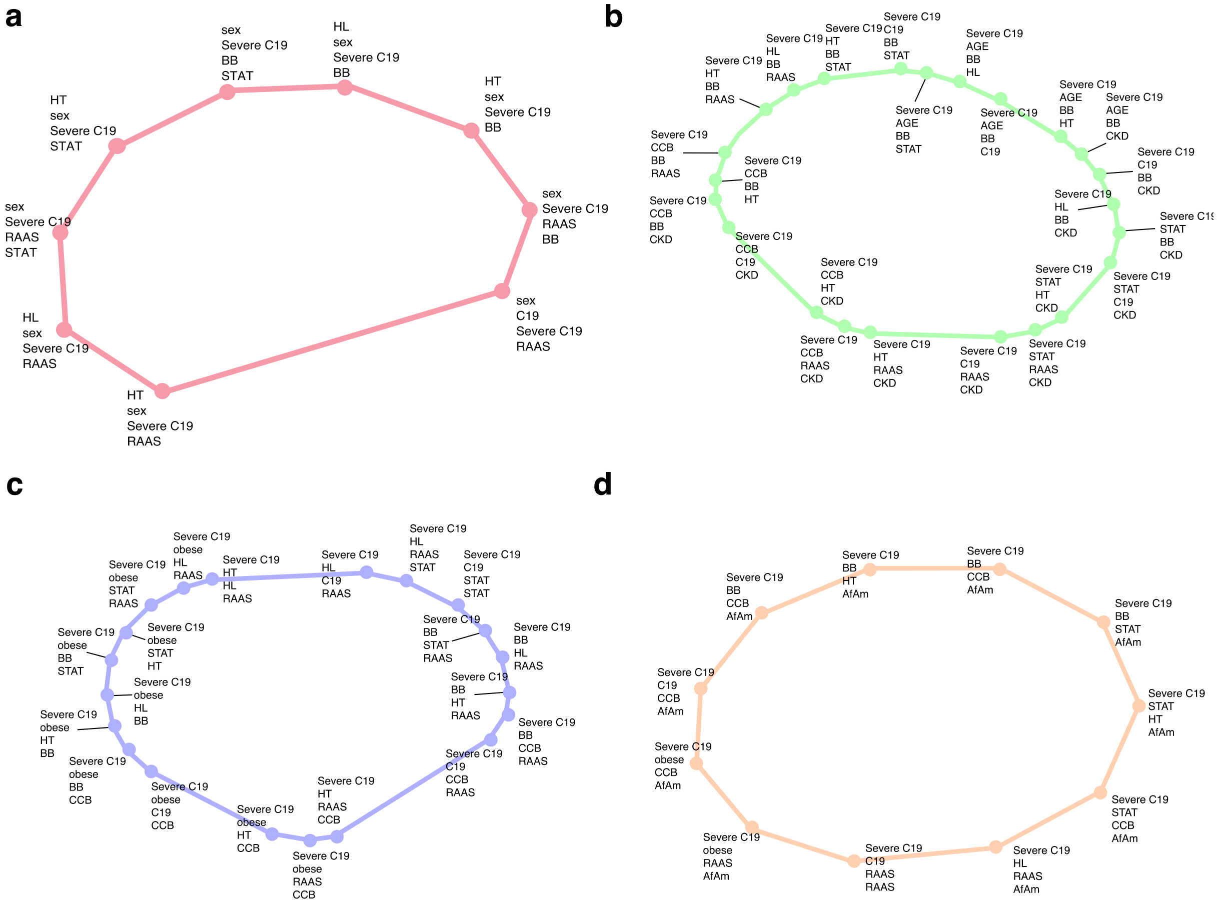

Supplementary Figure 3. Representative cycles corresponding to side-bar color codes in Figure 3A and Supplementary Figure 1 selected to highlight inclusions involving severe C19 and three HT drugs (ACE inhibitors, beta blockers, and ARBs), as well as African Americans. a) ACE inhibitor cycle, b) CCB/CKD/age cycle, c) CCB/obesity/HL cycle, and d) African American cycle.

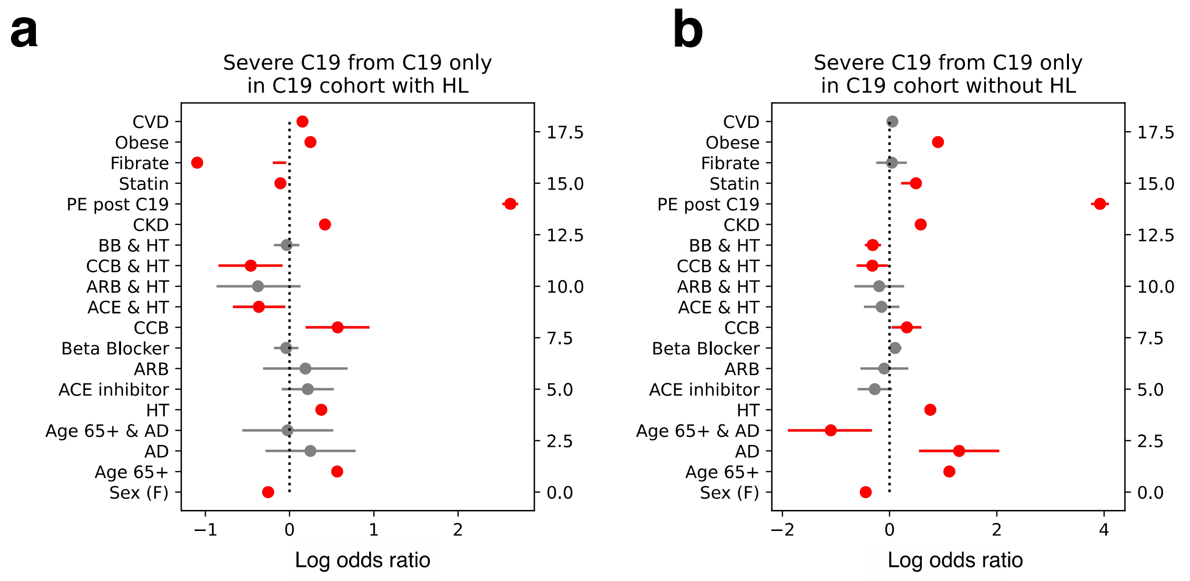

Supplementary Figure 4. Stratified logistic regression predicting severe C19 computed on C19 only patients from the C19 cohort with (a) and without (b) hyperlipidemia. Log odds ratios are plotted with 95\% confidence intervals and colored red if p-value < 0.05, otherwise gray.

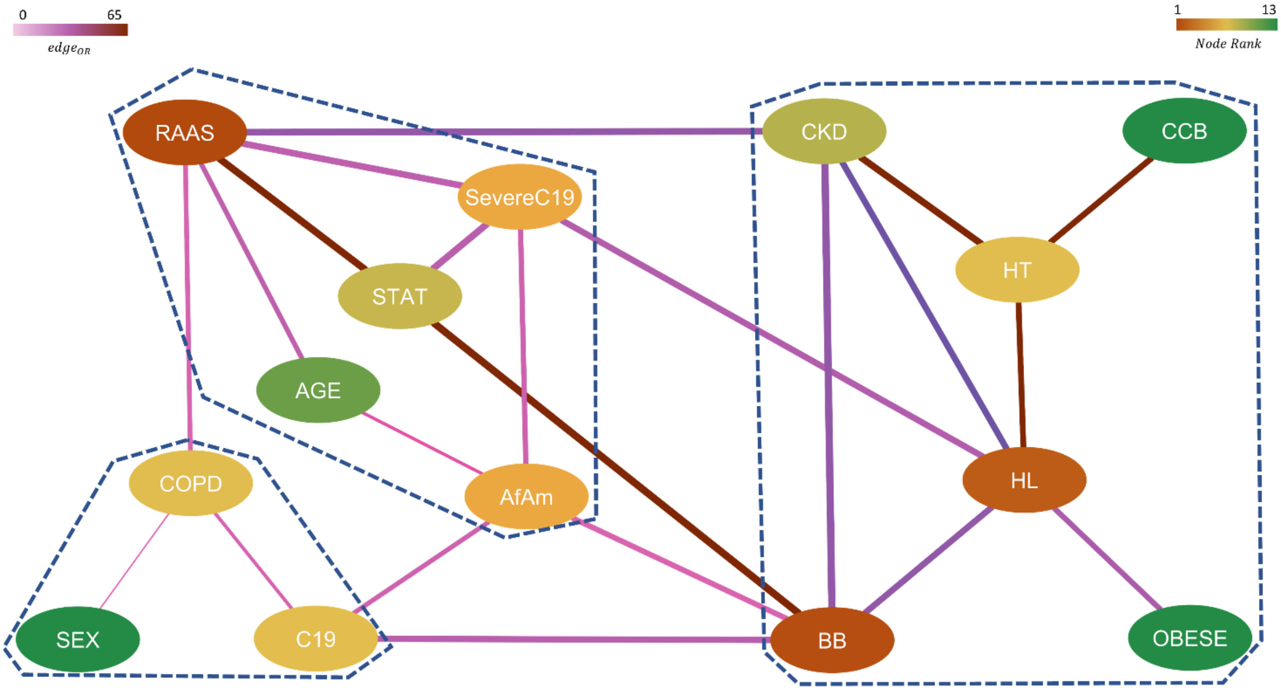

Supplementary Figure 5. Network representing significant, higher-order interactions between different features with nodes colored by their relative rank (gradient of brown to green corresponds to higher to lower rank) and edges colored by their respective pairwise odds ratios (gradient of light to dark corresponds to low to high OR) and edge width indicates the strength of the connection between them. The communities obtained from this network are marked by dashed lines.
